# Restrictions on antimicrobial use in aquaculture and livestock, Viet Nam

**DOI:** 10.2471/BLT.22.289187

**Published:** 2023-02-01

**Authors:** Juan J Carrique-Mas, Le Thi Hue, Le Thi Dung, Nguyen Thu Thuy, Pawin Padungtod

**Affiliations:** a Food and Agriculture Organization of the United Nations, Emergency Center for Transboundary Animal Diseases, Room 402, B3 Building, Van Phuc Diplomatic Compound 298, Kim Ma, 100000 Hanoi, Viet Nam.; b Department of Animal Health, Ministry of Agriculture and Rural Development, Hanoi, Viet Nam.

The World Health Organization (WHO) considers antimicrobial resistance one of the top 10 global health emergencies affecting humanity;^1^ antimicrobial use in animal production is one of its key drivers. Globally, animal production accounts for about three quarters of total antimicrobial use.^2^ In Viet Nam, approximately 2751 tonnes of antimicrobials are used in animal production annually,^3^ with pig and poultry production using the highest amounts.^4^ Levels of antimicrobial resistance in foodborne zoonotic pathogens (that is, non-typhoidal *Salmonella*) and antimicrobial residues in food products are also high in the country.^5^

In Viet Nam, exact data on quantities of antimicrobial consumption in animal production are unavailable, and information is limited to point surveys in defined geographical areas.^6^ Previous research suggested that prophylactic antimicrobial use practices were and are still widespread in chicken and pork production.^7^^,^^8^ For several decades, all veterinary medicine products had to be registered with the animal health department. Over 5000 antimicrobial-containing products are currently authorized in the country. A newly authorized product remains so for a five-year period. In Viet Nam, antimicrobials intended for animal production have a dedicated supply chain. Farmers have been able to purchase veterinary products over the counter in one of the 15 000 licensed veterinary drug shops. Virtually no antimicrobial-containing products for human use exist in food animal production, partly due to veterinary antimicrobials being extremely affordable.^9^

Under the umbrella of the tripartite framework of the Food and Agriculture Organization, the World Organisation for Animal Health and WHO, Viet Nam has developed a policy framework aimed at improving antimicrobial stewardship in livestock production and aquaculture, framed under national action plans (2017–2020 and 2021–2025).^10^^,^^11^ Recent legislation includes restrictions on antimicrobial use, and a roadmap for banning certain types of such use, in line with WHO recommendations. Here we provide a summary of the timelines for these restrictions alongside recommendations to support implementation of these regulations in Viet Nam, in addition to recommendations on specific activities to strengthen their implementation. 

## National restrictions

Box 1 provides a summary of recent regulations including restrictions on antimicrobial use in terrestrial animal and aquaculture production in Viet Nam, and their timelines. From the point of view of the end-user (that is, farmers, companies and veterinarians), three important restrictions exist. First, bans on antimicrobials in aquaculture feeds since May 2017, and on antimicrobial growth promoters in terrestrial animal production since January 2018. Second, a ban on the use of antimicrobials for disease prevention in mature terrestrial animals since March 2020, alongside a roadmap for a complete ban on prophylactic use in young terrestrial animals by 2026 (Decree 13/2020/ND-CP). Third, a roadmap with bans on the use of certain types of antimicrobials for prophylactic purposes based on their human health importance according to WHO, starting with critically important antimicrobials from January 2021. Furthermore, a prescription for prophylactic and therapeutic antimicrobial use is required since December 2020, depending on farm scale (terrestrial animals) and aquatic species raised.

Box 1 **Summary of regulations issued in Viet Nam including restrictions on antimicrobial use in animal production and their timelines**

***31 May 2016: Restrictions on the use of antimicrobial growth promoters in feed (6/2016/TT-BNNPTNT; circular legislation):***

- feed formulations may contain a maximum of two antimicrobial active ingredients. If two, it must be accompanied by scientific justification (from 15 July 2016)
- premix formulations must contain ≤ 20% strength in antimicrobials (from 15 July 2016)
- ban on antimicrobial growth promoters in livestock feeds (from 1 January 2018)

***4 April 2017: Management of livestock and aquatic feed (39/2017/ND-CP; decree)***

- inclusion of antimicrobials in feeds for aquaculture (any purpose) is not allowed (from 20 May 2017)
- announcement of a ban on prophylactic antimicrobial use in young livestock and poultry feeds (from 1 January 2021)
- antimicrobials in feed only can be added if supported by prescription issued by animal disease professionals as prescribed in the Law on Animal Health (from 20 May 2017)

***19 November 2018: Law on animal husbandry (32/2018/QH14; Law): Ban of non-approved antimicrobials to be included in feeds (from 1 January 2020)***

***21 January 2020: Elaboration of the Law on Animal Husbandry (13/2020/ND-CP, decree):***

- conditions for the authorization of prophylactic antimicrobial use based on stage of maturity: Piglets ≤ 25 kg or ≤ 60 days old; chickens, ducks, Muscovy ducks and quails ≤ 21 days-old; Rabbits ≤ 30 days-old; Buffaloes and calves ≤ 6 months (from 5 March 2020)
- roadmap for a ban on antimicrobial use for disease prevention by antimicrobial category based on their importance for human medicine, based on WHO criteria
- critically important antimicrobials banned from 1 January 2021: “Very important” from 1 January 2022; “Important” from 1 January 2023; “Others” from 1 January 2026

***9 November 2020: Prescription circular (12/2020/TT-BNNTPTNT):***

- compulsory prescription for antimicrobial use in livestock by farm scale: Large-scale from 25 December 2020; Medium-scale from 1 January 2022; Small-scale from 1 January 2023; Household from 1 January 2025
- compulsory prescription for antimicrobial use in aquaculture (treatment): Intensive (key species) from 25 January 2020; Intensive (non-key species) from 1 January 2023; Other types from 1 January 2025
- AMU prescriptions must be based on examination/diagnosis/testing results, except for prophylactic use (when allowed). Only veterinary drugs licensed in Viet Nam are allowed (from 25 December 2020)
- requirements for veterinary prescribers: Holding a professional veterinary certificate or bachelor’s degree in veterinary medicine with a practitioner license on animal disease diagnosis, examination, treatment, surgery and testing (from 25 December 2020)

Note: Definition of household, small, medium and large is based on number of livestock units as defined in Article 21 (Annex V) of Decree 13/2020/ND-CP.

## Comparison with other countries

In Viet Nam, antimicrobial growth promoters are not allowed in animal feeds, as is the case in the European Union (EU). Also following the EU model, from 2025 a prescription will be required for antimicrobial use in animal production in any circumstance. The Vietnamese roadmap will gradually ban prophylactic antimicrobial use for most antimicrobials – based on WHO criteria and starting with critically important antimicrobials for human health – with a full ban of any prophylactic antimicrobial use from 2026. In the EU, however, prophylactic usage is possible if supported by a veterinary prescription. In Canada, the inclusion of medically important antimicrobials (roughly corresponding to the WHO critically important, highly important and important categories) as antimicrobial growth promoters is banned, while still leaving a small number of allowed active ingredients. Furthermore, since 2018 a veterinary prescription is required for all medically important antimicrobials. In the United States of America, restrictions on antimicrobial growth promoters in feed and prescription requirements are voluntarily enforced by food animal production companies. Non-medically important antimicrobials in animals (roughly corresponding to the WHO medically important antimicrobials, highly important and important categories) will require a veterinary prescription from June 2023.

## Recommendations

First, we recommend the Vietnamese government to disseminate the regulations and monitor compliance. Effective compliance with these new regulations requires farmers and prescribers to be able to correctly identify antimicrobial veterinary products and their WHO category, understand the concept of prophylactic use, and be able to classify their farming enterprise depending on the number of livestock units. Doing so will necessarily require considerable dissemination efforts to reach a diverse landscape of stakeholders – ranging from large companies, veterinarians and pharmacists to farmers. Viet Nam has a very large farming population with a predominance of small-scale farms and household units. For example, in 2021, chickens raised in small- and medium-scale (that is, non-industrial) farms represented 61% of total chicken meat output (0.92 million of 1.49 million tonnes); of 2.05 million pig farms in the country, 1.70 million (83%) have less than 10 pigs.^12^ Veterinary authorities will need to make increased efforts to convey the contents of these regulations to small and household farms. Information materials aiming to convey the contents of these regulations in plain language to all veterinary pharmacies have already been developed, and are currently being distributed by the veterinary authorities to pharmacies and farmers, starting with the larger ones. In addition, the animal health department has planned training workshops delivered as roadshows, targeting veterinarians and drug shop owners.

We are aware that achieving full compliance with the new regulations will be challenging. Different agencies and departments within the government are discussing implementation and enforcement mechanisms. These mechanisms are currently within the legal competence of the provincial veterinary authorities. Formal assessment of compliance using knowledge, attitudes and practice studies in relevant production sectors in representative geographical locations are in the early stages of planning.

Second, manufacturers and distributors of antimicrobial-containing products should ideally label their products to encourage farmers’ compliance with the new regulations. 

A third recommendation is to assess impact on antimicrobial use and antimicrobial resistance. Ultimately, the aim of these regulations is to reduce levels of antimicrobial use and antimicrobial resistance in animal production. Changes in levels of antimicrobial use, as well as on antimicrobial resistance in commensal organisms and foodborne pathogens in animals, should be assessed using appropriate surveillance tools. With the support of international donors, the animal health department has been conducting surveillance on prevalence and antimicrobial resistance in *E. coli* and non-typhoidal *Salmonella* from pigs and poultry in 2017–2022. Investigating what effect, if any, these regulations are having on antimicrobial resistance patterns in these organisms will be relevant, although securing long-term funding is challenging. Monitoring impact on antimicrobial resistance in animal pathogens is, however, another long-term goal that will require additional investments in diagnostic and veterinary services.

The requirement of a prescription offers an opportunity for data collection on antimicrobial use, categorized by animal species. Data could potentially be collected through electronic prescription tools, and these data could feed back to Viet Nam’s antimicrobial use surveillance programme. The Vietnamese government is focusing first on electronic registration of antimicrobial-containing products.

The fourth and last recommendation is to engage with the industry and monitor impact on levels of disease. Many transnational food animal production companies are increasing their presence in Viet Nam as in other countries in south-east Asia. Many already have good private veterinary services and are implementing stewardship programmes to reduce unnecessary antimicrobial use. For such companies, a certain degree of harmonization of antimicrobial use restrictions across countries would be desirable, and this would help creating synergies to improve the responsible use of antimicrobials across the region. Keeping watch over any potential negative impact of the regulations on the incidence of disease and production in these farming systems would also be important.

## Conclusions

The recently issued regulations on antimicrobial use restrictions in livestock and aquaculture in Viet Nam places the country at the forefront of efforts among low- and middle-income countries in south-east Asia. At the end of its roadmap in 2026, antimicrobial use restrictions in the country will be the same as those of Canada and the EU countries. We advocate for a maximum compliance to these legislations and that these legislations should be reviewed and refined periodically. Implementation is expected to be particularly challenging in the numerous small-scale farming units. The recently issued Prescription Circular (Circular No. 12/2020/TT-BNNTPTNT) provides an opportunity for obtaining information on antimicrobial use at farm level – which would not have been possible before this legislation. Importantly, monitoring to what extent these regulations lead to measurable reductions in antimicrobial use in animals, and antimicrobial resistance in farms and the community will be useful. Since much of the farming landscape in Viet Nam is like that of other countries in south-east Asia, experiences and lessons learnt should be shared after the full roll-out of these legislative restrictions.
